# Review on the Bioaccumulation, Trophic Magnification, and Aquatic Toxicology of PFAS: Perspectives from Molecular Structure

**DOI:** 10.3390/toxics14090739

**Published:** 2026-08-22

**Authors:** Xindi Ye, Wei Cai, Jing Chen, Yutian Jin, Zhiquan Liu

**Affiliations:** Zhejiang Provincial Key Laboratory of Wetland Intelligent Monitoring and Ecological Restoration, School of Engineering, Hangzhou Normal University, Hangzhou 310018, China

**Keywords:** per- and polyfluoroalkyl substances (PFAS), carbon-chain lengths, functional groups, acid form and salt form, aquatic environment

## Abstract

Per- and polyfluoroalkyl substances (PFAS) are widely detected in aquatic environments, and their bioaccumulation, trophic magnification, and ecotoxicity have raised increasing concern. However, critical knowledge gaps remain regarding how differences in their molecular structures influence these processes and the mechanisms underlying such effects. This review synthesizes the current knowledge of the physicochemical properties, bioaccumulation, trophic magnification, and toxicity of PFAS in aquatic environments, with particular emphasis on the effects of the carbon-chain length, functional-group type, and acid or salt form. The current evidence generally indicates that long-chain PFAS exhibit higher bioaccumulation, trophic magnification, and toxicity than short-chain PFAS, largely because of their enhanced hydrophobicity, stronger protein-binding affinity, and slower elimination rates. As two major PFAS subclasses, perfluoroalkyl sulfonates (PFSAs) generally show higher bioaccumulation and toxicity than perfluoroalkyl carboxylates (PFCAs). For example, the bioaccumulation factor of perfluorooctane sulfonate (PFOS) is approximately two- to three fold higher than that of perfluorooctanoic acid (PFOA). Some ether-containing PFAS, developed as safer alternatives, also exhibit concerning ecological hazards, particularly a high potential for trophic magnification. In addition, acid form PFAS generally accumulate more readily and induce more severe toxic effects than their corresponding salt forms. These findings highlight the need to further improve structure-based frameworks for PFAS ecological risk assessment.

## 1. Introduction

Per- and polyfluoroalkyl substances (PFAS) comprise a diverse class of synthetic organofluorine compounds that typically contain one or more highly fluorinated carbon moieties [1]. Their unique combination of fluorinated hydrophobic or oleophobic chains and hydrophilic polar head groups confers exceptional surface activity, interfacial partitioning behavior, and chemical stability. As a result, PFAS have been extensively incorporated into firefighting foams, nonstick coatings, water- and oil-repellent textiles, food packaging materials, metal-plating processes, and numerous industrial and consumer products [2,3]. The global production, use, and continuous release of PFAS have made them contaminants of major environmental concern. Because of their high persistence and mobility, PFAS can be readily transported through surface water, groundwater, and drinking water systems. In aquatic environments, organisms are exposed to PFAS through water, sediment contact, and dietary intake. Their subsequent uptake and accumulation increase internal concentrations and may contribute to adverse biological effects and the disruption of aquatic ecosystem homeostasis [4,5,6]. Legacy PFAS, particularly perfluorooctanoic acid (PFOA) and perfluorooctane sulfonate (PFOS), have been listed as persistent organic pollutants under the Stockholm Convention and identified as priority substances of concern by the United Nations Environment Programme and the U.S. Environmental Protection Agency [7].

Previous reviews have provided valuable syntheses of PFAS occurrence, environmental transport, analytical methods, degradation technologies, ecological toxicity, and toxicological mechanisms across environmental matrices and biological tissues [8,9,10,11,12]. However, the extent to which structural attributes control PFAS bioaccumulation, tissue distribution, trophic transfer, and toxicity in aquatic organisms remains insufficiently resolved. In particular, the roles of the carbon-chain length, functional-group identity, and the acid or salt form of PFAS have not been systematically integrated from an aquatic ecotoxicological perspective. Broad classifications such as “legacy PFAS”, “emerging PFAS”, or “short-chain alternatives” may obscure important differences in the environmental behavior and biological effects among structurally distinct PFAS, which may limit the accuracy of ecological risk assessment and the rational design of safer PFAS alternatives.

Previous reviews have primarily focused on individual aspects of PFAS, such as their occurrence, environmental fate, bioaccumulation, or toxicity, or have examined only their chain-length effects or functional-group-dependent differences. This review integrates the carbon-chain length, functional-group identity, and the acid or salt form of PFAS within a unified structure-dependent framework, and the relationships between PFAS structural characteristics and aquatic ecotoxicity are examined. Emphasis is placed on four key aspects: (i) toxic effects and potential mechanisms induced by PFAS in aquatic animals; (ii) chain-length-dependent differences in physicochemical properties, bioaccumulation potential, food-web transfer, and toxicity; (iii) functional-group-dependent patterns of tissue distribution and toxicological responses in aquatic organisms; and (iv) differences in bioaccumulation, intergenerational transfer, and toxicity between acid and salt form PFAS. This integrated perspective may improve structure-based ecological risk assessment and support the identification of safer PFAS alternatives.

## 2. Methods

A literature search was conducted in PubMed and the Web of Science Core Collection for publications from 1 January 2000 to 31 July 2026. Search terms covered PFAS, aquatic organisms, bioaccumulation, bioconcentration, tissue distribution, trophic magnification, biomagnification, and major toxicological endpoints, including hepatotoxicity, neurotoxicity, and developmental and reproductive toxicity. Only peer-reviewed English-language journal articles were considered. Eligible publications included original research articles, reviews, and relevant synthesis or conceptual articles, whereas conference abstracts, editorials, and book chapters were excluded. Studies were considered directly relevant when they investigated PFAS in aquatic organisms, including fish, amphibians, and aquatic invertebrates, and reported outcomes related to bioaccumulation, tissue distribution, trophic transfer, or toxicity, particularly in relation to carbon-chain length, functional-group identity, or acid/salt form. Studies focusing solely on abiotic occurrence, analytical methods, remediation, treatment, or degradation were excluded from the core synthesis. Publications were also excluded when the PFAS compound/class, study species or experimental model, exposure or environmental context, or relevant biological or toxicokinetic outcomes could not be clearly identified. In addition to the core aquatic-organism literature, selected physicochemical, toxicokinetic, protein-binding, receptor-binding, and other mechanistic studies were included as supporting evidence when they provided information relevant to explaining structure-dependent differences among PFAS. Evidence derived from non-aquatic experimental systems was used only to support mechanistic interpretation and was distinguished from direct evidence obtained from aquatic organisms. After title, abstract screening and, where necessary, full-text assessment, 113 publications were retained.

## 3. Toxic Effects and Biological Mechanisms

**Figure 1:**
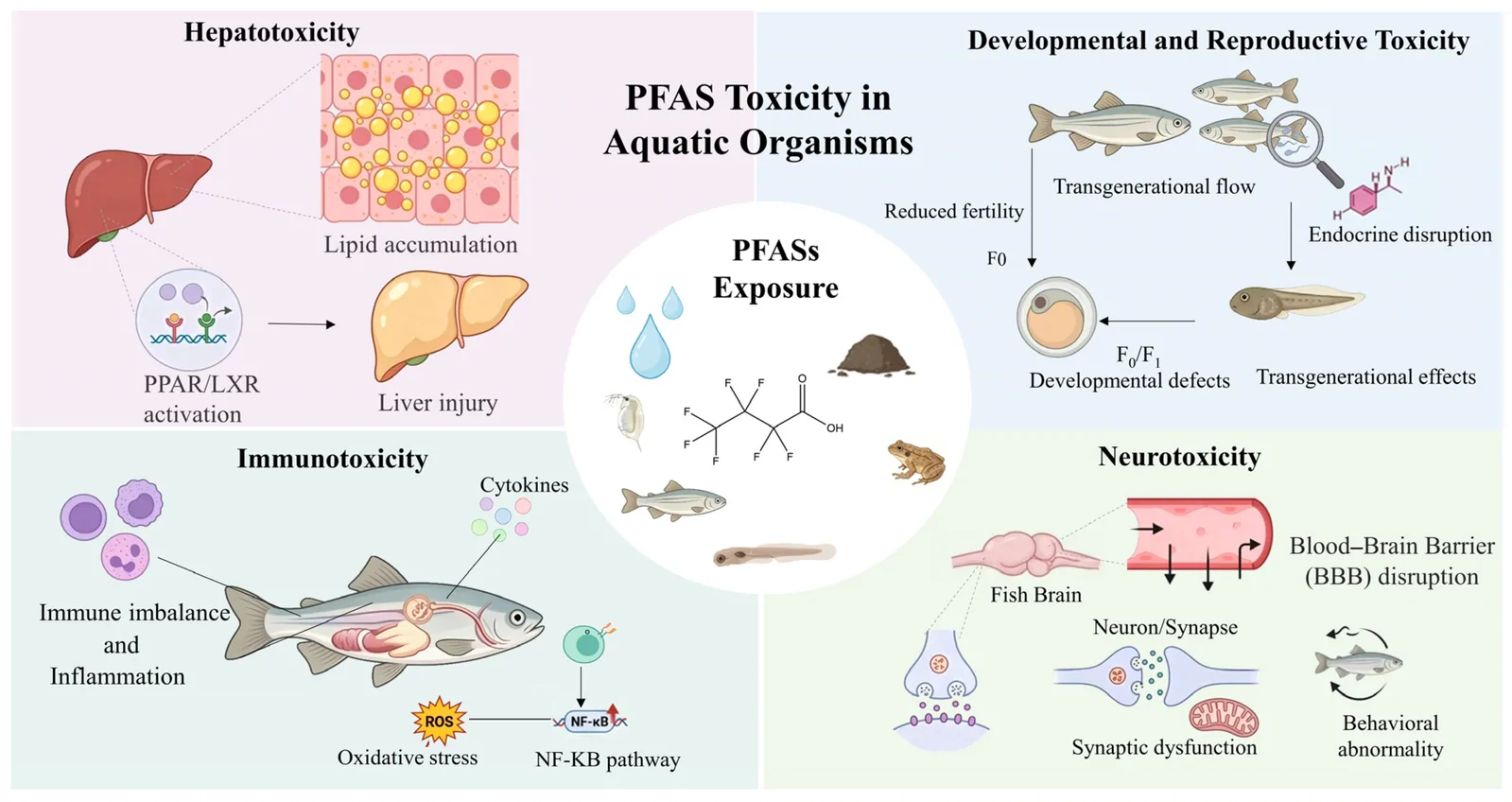
Study of the toxic effects of per- and polyfluoroalkyl substances (PFAS) on different aquatic organisms.

PFAS can accumulate in aquatic organisms following exposure through water or sediments, and they can also be transferred through food chains and food webs, thereby increasing internal exposure. Once taken up, PFAS may disrupt physiological and biochemical processes, resulting in hepatotoxicity, developmental and reproductive toxicity, endocrine disruption, immunotoxicity, neurobehavioral toxicity, genotoxicity, and epigenetic effects [13,14,15]. Accordingly, this section focuses on hepatotoxicity, developmental and reproductive toxicity, immunotoxicity, and neurotoxicity, because they represent major well-documented adverse outcomes across key physiological systems and provide a mechanistic basis for the subsequent comparison of structure-dependent PFAS effects. We acknowledge that our review on the toxicity of PFAS to aquatic organisms is not exhaustive, and we provide an overview of what is available, focusing on some examples that highlight the diversity of effects being reported [16,17,18] (Figure 1).

### 3.1. Hepatotoxicity

Exposure to PFAS, such as PFOS and its alternative 6:2 Cl-PFESA, alters fatty acid, glycerophospholipid, and steroid metabolism. Both compounds can also perturb peroxisome proliferator-activated receptor (PPAR) signaling at environmentally relevant concentrations. They alter the expression of genes involved in fatty acid synthesis, fatty acid β-oxidation, and cholesterol transport, leading to the enrichment of fatty acids and total cholesterol from the blood to the liver and accelerating fatty acid esterification and cholesterol conversion to steroids, which ultimately induces hepatic lipid accumulation and vacuolization pathology and damage, with sex differences in the degree of metabolic disturbance [19]. For example, PFAS accumulated in the tissues of black-spotted frogs near the fluoridation site, and the liver concentrations were positively correlated with the hepatosomatic index (HSI), triglyceride (TG), and total cholesterol (TC) levels. Controlled exposure experiments further showed hepatic lipid deposition, hepatocellular hypertrophy, elevated serum lipid levels, a 1.33–1.87-fold increase in hepatic TG, and altered hepatic fatty acid composition. PFAS upregulated the expression of lipid synthesis-related genes (e.g., *fasn*, *acc1*, *dgat*) and downregulated genes involved in fatty acid β-oxidation (e.g., *cpt1*, *acox1*) by directly binding to and activating liver X receptor alpha (LXRα) and PPARα, ultimately leading to lipid metabolic disorders [20,21]. Exposure to low concentrations of PFHxA disrupted the intestinal physical and microbial barriers in *Larimichthys crocea*, subsequently inducing severe hepatic inflammation and cell death via the gut–liver axis [22]. Developmental exposure of African clawed frogs (*Xenopus laevis*) to environmentally relevant concentrations of PFAS perturbed lipid metabolism in a sex-specific manner and altered hepatic lipid composition; these effects were potentially mediated independent of the classic PPARα pathways [23]. Both conventional and alternative PFAS have been detected at high concentrations in liver tissues, and these concentrations have been associated with changes in lipid-metabolism indicators. PFAS regulate downstream lipid synthesis and catabolism-related genes through the activation of the nuclear receptor pathway (PPARα, LXRα, or PPARγ), leading to alterations in hepatic fatty acid composition, liver injury, and serum-hepatic lipid transport imbalance. Collectively, these studies indicate that PFAS may disrupt hepatic lipid homeostasis by altering lipid transport, synthesis, oxidation, and tissue deposition. However, the underlying pathways appear to vary among compounds, species, and exposure conditions, involving both nuclear receptor-dependent and alternative mechanisms such as gut–liver interactions. Standardized comparative studies are needed to clarify the links between PFAS exposure, molecular responses, and adverse liver outcomes.

### 3.2. Developmental and Reproductive Toxicity

Altered reproductive phenotypes, reduced fertility and fecundity, and dysregulation of key genes controlling reproduction can lead to altered population dynamics in aquatic organisms, ultimately affecting ecosystem health. Gasparini et al. [24] found that while the traditional perfluoroalkyl substance PFOA and its novel alternative GenX did not significantly affect guppies’ survivorship and body size metrics, GenX significantly altered male courtship behavior patterns and increased the courtship frequency. PFAS significantly reduced sperm motility, and PFAS exposure induced the differential expression of genes related to immunomodulation, oxidative stress, extracellular matrix function, and apoptosis in the testis, according to transcriptome analysis. The larval-stage exposure of gray treefrogs to environmentally relevant concentrations of PFAS induced carry-over effects on terrestrial growth and interacted with exposure to the fungal pathogen Batrachochytrium dendrobatidis (Bd), with the replacement compound GenX exerting more pronounced effects on post-metamorphic performance than its legacy counterpart PFOA [25]. Both the short-chain perfluorinated and polyfluorinated alkyl substances PFBS and FBSA were significantly more toxic in multiple generations of exposure: the ecotoxicity of PFBS increased 1.8- to 3.0-fold by the sixth generation (F_5_) compared to the initial generation (F_0_), whereas the increase in toxicity of FBSA was 3.6- to 6.4-fold. The toxicity enhancement of PFBS was mainly due to progressive compound accumulation across generations, while FBSA showed specific reproductive toxicity, and its toxicity deterioration was closely related to the physiological damage across generations [26]. Hence, PFAS systematically harms the reproductive and developmental health of aquatic organisms by disrupting their endocrine systems, leading to an imbalance in the ratio of sex hormones to thyroid hormones, affecting their development, and leading to multigenerational effects.

### 3.3. Immunotoxicity

The immune system is the key defense against pathogens and also plays an important role in maintaining the homeostasis of physiological processes such as development, growth, inflammatory response, and tissue repair. The immune system is a sensitive target of PFAS, and evaluating the immune effects of PFAS is an important strategy to examine their safety [27]. The traditional perfluorinated compounds PFOA, PFOS, and their alternatives hexafluoropropylene oxide dimer acid (HFPO-DA) and perfluoroethylcyclohexane sulfonate (PFECHS) induced immunotoxicity in zebrafish embryos at environmentally relevant concentrations. PFAS exposure led to an increase in the number of neutrophils and a significant increase in the levels of the pro-inflammatory factors TNF-α, IL-1β, IFN-α, complement C3, and nitric oxide (NO), while it decreased lysozyme activity, caused an imbalance of immune homeostasis, and activated an inflammatory response. PFAS upregulated key genes such as MyD88, TRAF6, and NF-κB through the activation of the TLR, NLR, and RLR signaling pathways, which in turn drove the downstream MAPK/NF-κB inflammatory pathway [28]. The PFAS subclass PFAA mixture and high concentrations of PFOS triggered similar proteomic perturbations in rainbow trout plasma, significantly activating the PPAR δ/γ pathway and affecting lipid metabolism, oxidative stress, and neurological-related proteins; head kidney tissues were the most sensitive to exposure to PFOA and high concentrations of the mixture, with PFOA exposure specifically up-regulating interferon alpha (IFNA) production, and high concentrations of the mixture synergistically induced elevated TLR3/7 expression, accompanied by the inhibition of antioxidant enzymes such as catalase (CAT). Hence, PFAA poses a comprehensive risk to aquatic organisms by inducing a cascade of oxidative stress and inflammation that impairs the immune function of the head kidney, thereby inducing immunotoxicity [29].

### 3.4. Neurotoxicity

PFAS may cross the blood–brain barrier (BBB) through transmembrane diffusion or transport processes involving serum albumin and fatty acid-binding proteins. Once present in neural tissues, PFAS may disrupt endothelial tight junctions, neurotransmitter levels, and synaptic calcium homeostasis, thereby contributing to neurotoxicity [30,31,32]. Developmental exposure to environmentally relevant concentrations of PFAS resulted in PFAS accumulation and complex neurotransmitter alterations in the brains of Northern leopard frogs, most notably with a marked increase in acetylcholine levels [33]. Exposure to environmental concentrations of PFOS, PFOA, and PFHxA significantly reduced comfort behaviors such as resting and group swimming frequency in crucian carp (*Carassius auratus*), inhibited brain tissue acetylcholinesterase AChE activity and dopamine levels, and induced pathological brain damage, while PFAS led to an imbalance of oxidative stress in the brain and activated inflammatory responses and cellular stress, triggering behavioral abnormalities and neurotoxicity [34]. PFOS alternatives F-53B and sodium p-perfluorononenoxybenzene sulfonate (OBS) interfered with amino acid neurotransmitter metabolic pathways in zebrafish, disrupting the blood–brain barrier structure, inhibiting the formation of cilia in ventricular membrane cells, and blocking the Wnt signaling pathway, which induced ventricular enlargement in the midbrain and an imbalance in dopamine secretion, thereby affecting circadian rhythmic behaviors [35]. Wang et al. [36] found that PFECHS neurotransmitter metabolism was disturbed after exposure to marine medaka (*Oryzias melastigma*), and the level of γ-aminobutyric acid (GABA) in the brain was decreased in a concentration-dependent manner, while the activities of AChE and monoamine oxidase (MAO) were significantly elevated. PFECHS disrupts the function of GABAergic and glutamatergic synapses through the downregulation of the expression of GABA synthase and transporter proteins, GABA receptor, and glutamatergic receptors and transporter proteins in the brain and interferes with calcium signaling pathways, leading to impaired synaptic transmission and causing behavioral abnormalities. Exposure to PFAS is closely related to nerve damage and effects on brain function and behaviors. Disruption of the BBB integrity, perturbation of neuronal and glial cell function, NF-kB-mediated activation of inflammatory vesicles, and apoptosis due to the dysregulation of mitochondrial calcium homeostasis are some of the ways in which PFAS contribute to the neurotoxicity of aquatic organisms either directly or indirectly.

The above evidence indicates that PFAS can induce multiple toxic effects in aquatic organisms. However, these effects are not determined solely by the exposure concentration, exposure duration, or species sensitivity. Increasing evidence suggests that the environmental behavior and biological toxicity of PFAS are largely regulated by their molecular structures, including variations in their carbon-chain length, functional groups, and chemical forms. Therefore, differences in the physicochemical properties, bioaccumulation behaviors, toxic effects, and potential mechanisms of PFAS with different chain lengths, functional groups, and chemical forms are compared below, and the potential mechanisms underlying structure-dependent differences in PFAS-induced toxicity are analyzed.

## 4. PFAS with Different Chain Lengths

### 4.1. Physicochemical Properties with Different Chain Lengths

Owing to their unique amphiphilic structures, which consist of a perfluorinated carbon chain and a hydrophilic functional group, the physicochemical properties of PFAS are largely governed by their carbon-chain length [1]. In this review, the carbon-chain length is expressed as the total number of carbon atoms in the molecule. Long-chain PFAS are defined as perfluoroalkyl carboxylic acids (PFCAs) containing ≥ 8 carbon atoms and perfluoroalkane sulfonic acids (PFSAs) containing ≥ 6 carbon atoms, while the remaining compounds are classified as “short-chain” [11].

**Figure 2:**
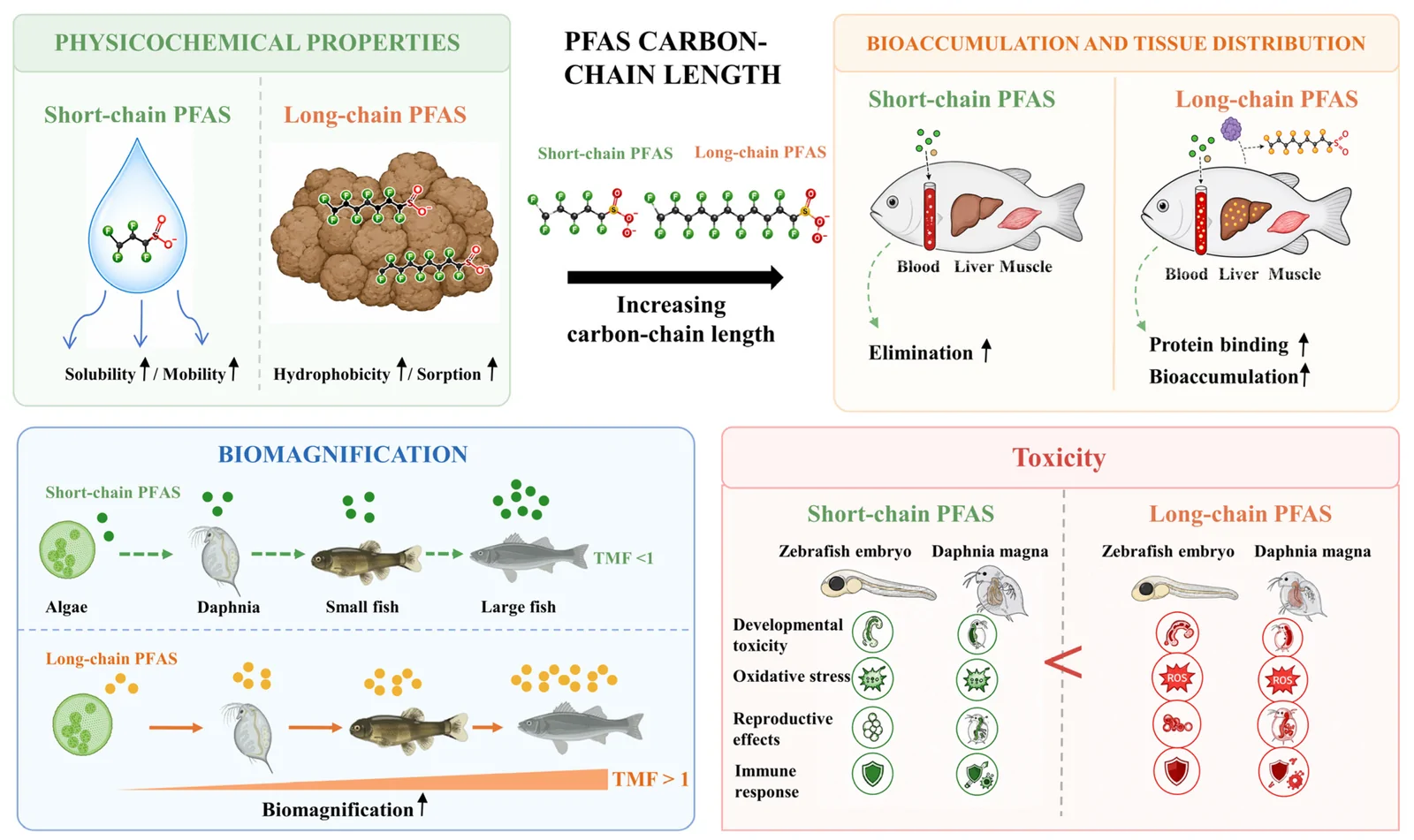
Effects of the carbon-chain length on the physicochemical properties, bioaccumulation, biomagnification, and toxicity of per- and polyfluoroalkyl substances (PFAS). Upward arrows indicates a higher level or stronger tendency.

The carbon-chain length has been shown to exert a pronounced influence on the water solubility and hydrophobicity of PFAS. As the carbon-chain length increases, the water solubility of PFAS is gradually reduced, and their hydrophobicity is markedly enhanced [37]. Short-chain PFAS, owing to their smaller molecular size and shorter hydrophobic tails, interact more strongly with water molecules and therefore exhibit higher water solubility and environmental mobility. Consequently, they are commonly present in aquatic environments in dissolved forms [38]. In contrast, long-chain PFAS, which possess larger fluorocarbon backbones and stronger hydrophobicity, are more likely to partition from the aqueous phase into sediments, organic matter, and biological tissues, thereby exhibiting higher environmental persistence [39,40]. In addition, PFAS also exhibit pronounced surface activity. Their molecular structures, composed of hydrophobic fluorocarbon tails and hydrophilic head groups, enable them to effectively reduce the surface tension of water and become enriched at interfaces [41]. This interfacial behavior is further strengthened with the increasing chain length. Long-chain PFAS, due to their higher hydrophobicity and stronger intermolecular interactions, are more readily enriched at air–water and solid–liquid interfaces through hydrophobic interactions [42]. Conversely, short-chain PFAS, because of their stronger hydrophilicity, tend to remain in the aqueous phase rather than accumulate at interfaces, thereby displaying higher mobility [43]. This pattern has been confirmed by environmental distribution studies [44]. Therefore, differences in the chain length may lead to variations in the physicochemical properties of PFAS, including their water solubility, hydrophobicity, and interfacial activity, which may further determine their environmental fate, bioaccumulation potential, and toxic effects [39] (Figure 2).

### 4.2. Bioaccumulation and Distribution with Different Chain Lengths

The bioaccumulation factor (BAF), defined as the ratio of the PFAS concentration in an organism to that in the surrounding water, is commonly used to quantify the bioaccumulation resulting from all relevant exposure routes [45]. Numerous studies have demonstrated that long-chain PFAS are generally accumulated in aquatic organisms to a significantly larger extent than short-chain PFAS. Such chain-length-dependent differences have been observed in fish [46], amphibians [47], and aquatic invertebrates [48].

**Figure 3:**
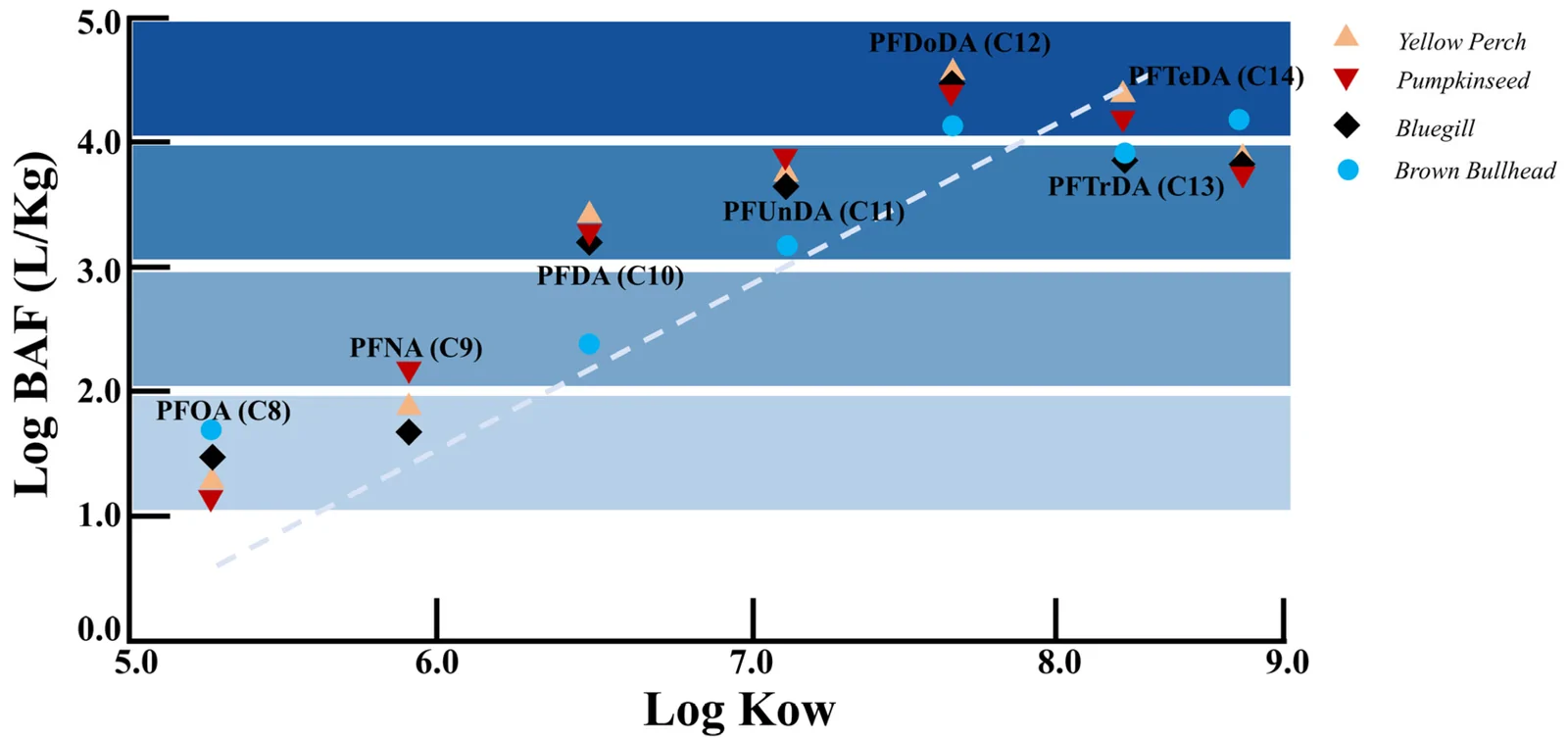
Bioaccumulation factor (BAF) and logarithm of the octanol–water partition coefficient (log K_o_w) of perfluorocarboxylic acids (PFCAs) in freshwater fish.

The mechanisms underlying these differences can be mainly attributed to three interacting toxicokinetic processes. First, as described above, long-chain PFAS generally exhibit stronger interfacial and membrane-partitioning capacities, which facilitate their transfer from the aqueous phase to biological membranes, body surfaces, and tissues. An increasing chain length may strengthen the interactions between the fluorinated tails of PFAS and the hydrophobic regions of phospholipid membranes, thereby promoting their uptake and retention at biological interfaces. Accordingly, log-transformed BAFs are generally positively correlated with the logarithm of the octanol–water partition coefficient (log K_o_w) (Figure 3), a pattern that has been reported in both aquatic vertebrates and invertebrates [48,49,50,51]. Second, unlike conventional POPs, which are commonly stored in lipid-rich tissues, PFAS tend to bind to proteins, such as serum albumin and fatty acid-binding proteins, because of their polar head groups [52]. The binding of PFAS to serum albumin is strongly chain-length-dependent, with longer-chain PFAS generally exhibiting higher protein-binding affinities [53]. This pattern may be attributed to stronger interactions between longer fluorinated tails and hydrophobic regions within protein-binding cavities [54]. Protein binding also helps to explain the preferential distribution of long-chain PFAS in protein-rich tissues, particularly blood and liver. For example, a survey of PFAS in fish from waterbodies in Massachusetts, USA, showed that PFAS concentrations were generally highest in plasma, followed by the liver and muscle, consistent with their affinity for blood proteins [55]. Similarly, PFAS burdens in fish tissues, including blood, gonads, and muscle, together accounted for more than 90% of the total body burden in these organisms [46]. Third, transporter-mediated uptake and reabsorption may contribute to the higher retention of certain long-chain PFAS. As amphiphilic organic anions, PFAAs can be recognized by transporters involved in the disposition of organic anions and bile acids, including OAT4, OATPs, NTCP, and ASBT. OAT4-mediated uptake was observed for PFHxS and PFOS but was negligible for the shorter-chain PFBS [56]. Similarly, several hepatic OATPs transported PFHxS, PFOS, and C8–C9 PFCAs more efficiently than PFBS or C7 PFCA [57]. Bile acid transporters may also promote intestinal reabsorption following biliary excretion [58]. These processes can reduce net elimination and prolong the retention of long-chain PFAS.

Consequently, stronger protein and tissue binding, together with transporter-mediated reabsorption and recirculation, may slow PFAS elimination and prolong their biological half-lives, ultimately contributing to chain-length-dependent bioaccumulation. Long-chain PFAS generally exhibit slower elimination rates and longer biological half-lives than their short-chain counterparts, resulting in higher internal persistence and cumulative body burdens. For example, the hepatic half-lives of PFOS, perfluorohexanesulfonic acid (PFHxS), and perfluorobutanesulfonic acid (PFBS) in adult rainbow trout were reported to be 20.4, 9.3, and 6.5 days, respectively, indicating progressively slower elimination with increasing PFSA chain length [59]. Similar chain-length-dependent differences in elimination have also been demonstrated in aquatic invertebrates. In *crustaceans*, long-chain PFAS generally exhibited significantly slower elimination rates, whereas short-chain PFAS were eliminated more rapidly. Overall, the higher bioaccumulation potential of long-chain PFAS compared with short-chain PFAS is likely driven by the combined effects of stronger interactions with biological membranes and proteins, transporter-mediated reabsorption and recirculation, and reduced net elimination rates.

### 4.3. Trophic Magnification Associated with Different Chain Lengths

**Figure 4:**
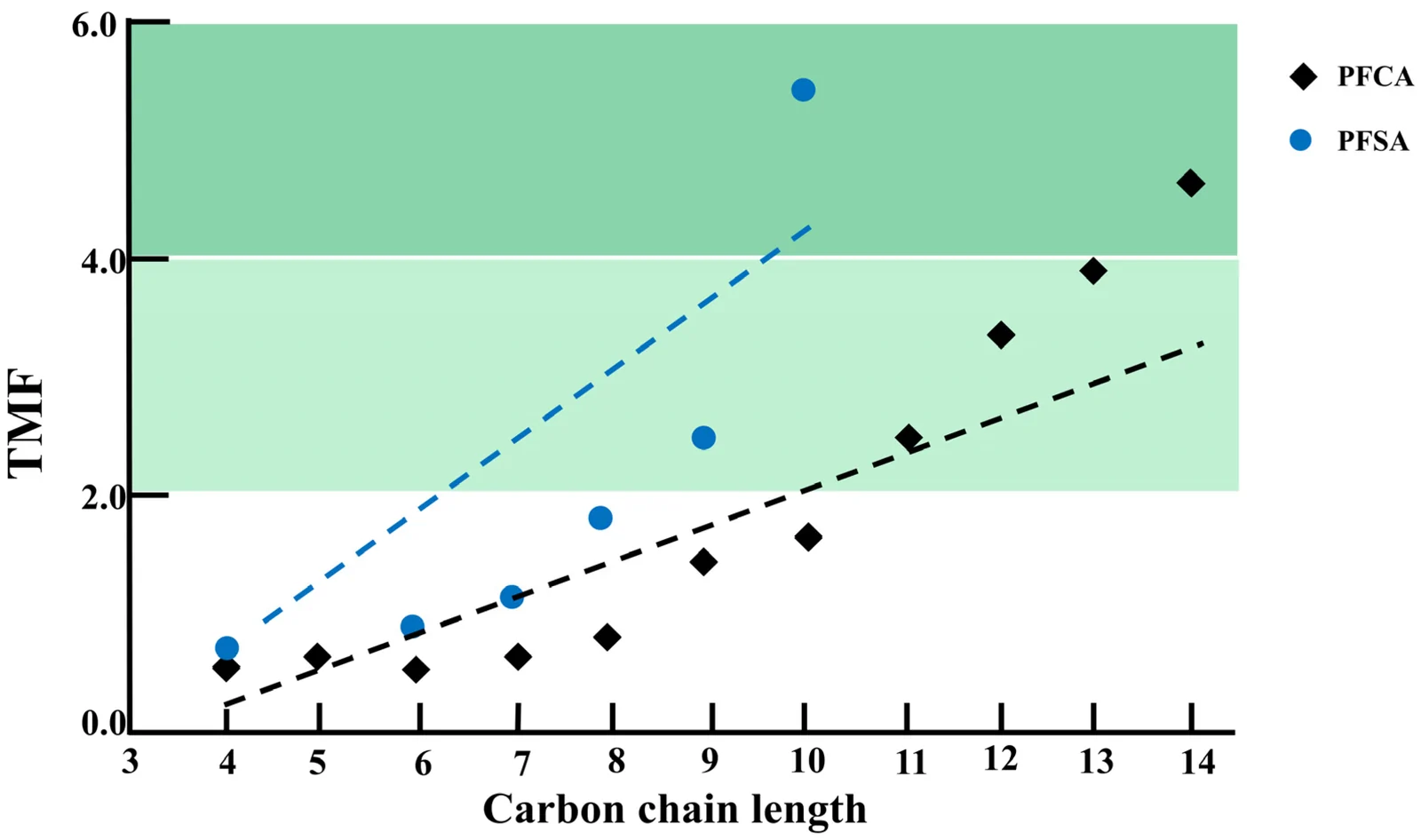
Trophic magnification factors (TMFs) of perfluorocarboxylic acids (PFCAs) and perfluoroalkane sulfonic acids (PFSAs) with different chain lengths in aquatic food webs.

Trophic magnification factors (TMFs) quantify changes in contaminant concentrations across trophic levels and, therefore, characterize food-web-mediated exposure [60]. A TMF higher than 1 indicates trophic magnification and potentially higher dietary exposure in predators, whereas a TMF less than 1 indicates trophic dilution. Overall, the biomagnification of long-chain PFAAs has been observed across entire food webs. In the Taihu Lake food web, perfluorohexanoic acid (PFHxA) and PFOA were found to contribute predominantly to the aqueous phase, whereas longer-chain PFAS, such as perfluorodecanoic acid (PFDA) and perfluoroundecanoic acid (PFUdA), were more prominently detected in biota. The TMFs of perfluorodecanoic acid (PFDA) and perfluorododecanoic acid (PFDoA) were reported to be 2.43 and 2.68, respectively, indicating that longer-chain PFAS were more readily magnified along the food chain [61,62] (Figure 4). Similar findings have also been reported in other studies, in which TMFs of 1.50 and 1.74 were determined for PFDA and PFUdA, respectively, further confirming the chain-length-dependent biomagnification potential of PFAS in food webs [63]. By contrast, more complex patterns have been observed for short-chain PFAS in food webs. However, the relationship between chain length and TMF is not simply linear [64]. Trophic dilution of short-chain PFAA was observed in higher-trophic-level taxa (TL > 3.50; TMF = 0.71), whereas biomagnification was detected in lower-trophic-level taxa (TL < 3.50; TMF = 2.19) [12,65]. This pattern may partly reflect the more rapid metabolism and elimination of short-chain PFAS in higher-trophic-level organisms. In lower-trophic-level organisms such as mollusks, benthic feeding and other ecological traits may increase exposure and accumulation [66]. In addition, among long-chain PFAS, TMFs may be substantially influenced by functional groups, bioavailability, and food-web structure, resulting in considerable variability. In some bay food webs, the trophic magnification of long-chain PFAS did not increase consistently with the carbon-chain length [62]. Collectively, these findings suggest that chain length is an important factor influencing PFAS biomagnification in biota, and further investigation is needed to elucidate the effects of other structural factors.

### 4.4. Toxicity Differences with Different Chain Lengths

Long-chain PFAS have generally been shown to exhibit higher toxicity than short-chain PFAS. For example, after zebrafish embryos were exposed to environmentally relevant concentrations of long-chain PFOS and reared to adulthood, their hepatic lipid contents were significantly higher than those in the PFBS-exposed group. A larger reduction in egg production was also observed, indicating the stronger hepatotoxic and reproductive effects of long-chain PFAS during zebrafish development. Moreover, PFAS-induced disruption of lipid metabolism was identified as a potential driver of reduced egg production, further suggesting the stronger integrated toxicity of long-chain PFAS [67]. The benchmark dose (BMD) represents the dose required to induce a measurable low-level toxic response. In a developmental toxicity assessment of 139 PFAS in zebrafish, the long-chain compound PFDA produced the most pronounced developmental effects. Its BMD_10_ value was 0.223 μM, which was much lower than those of most other PFAS, indicating a higher potential to induce body-axis malformations and behavioral alterations [68]. In addition, PFNA-induced immunotoxicity through the TLR pathway was significantly stronger than that induced by PFBA in zebrafish [69]. Similar patterns have been reported in invertebrates; the acute toxicity of C4–C6 PFCAs to *Daphnia magna* was positively correlated with the carbon-chain length [70].

The higher toxicity reported for long-chain PFAS may partly reflect the higher internal burdens caused by longer biological half-lives, lower clearance rates, and stronger plasma protein binding. Once PFAS reach target tissues, nuclear receptor activation may further contribute to chain-length-dependent toxicity, whereas penetration of biological barriers primarily affects delivery to those tissues. Increasing evidence indicates that PFAS-induced nuclear receptor activation is associated with the carbon-chain length. In vitro assays using COS-1 cells transiently transfected with human PPARα showed that PFAA-mediated receptor activation generally increased with the carbon-chain length, with this trend extending to C9 among carboxylic acids [71] (Table 1). This suggests that long-chain PFAS may bind nuclear receptors more readily and disrupt key physiological processes, including lipid metabolism, energy metabolism, and endocrine regulation [72]. However, these findings were obtained using a human receptor system, and the receptor sensitivity, ligand-binding affinity, and downstream responses may differ among aquatic species. Aside from PPARs, thyroid hormone receptors and reproductive hormone receptors are also affected by the chain length. Longer fluorocarbon chains can provide more hydrophobic interaction sites, thereby increasing the receptor-binding stability and biological activity [73]. Long-chain PFAS also tend to show more tissue penetration and maternal transfer, thereby increasing exposure during sensitive developmental windows. PFOS, for example, can accumulate in fish and amphibians and be transferred to offspring through yolk or maternal transport in viviparous species [74,75]. Consequently, increased embryonic and larval exposure may enhance the likelihood of developmental, neurobehavioral, and reproductive toxicity.

Overall, the carbon-chain length is an important factor influencing the environmental behavior, bioaccumulation, trophic magnification, and toxicity of PFAS in aquatic organisms. Long-chain PFAS generally exhibit higher bioaccumulation and toxicity. However, some short-chain PFAS may be more toxic than long-chain PFAS for specific endpoints. In a combined toxicity assessment using zebrafish, the short-chain compound PFHxA showed higher toxicity than the long-chain compound PFOA across several endpoints, including oxidative damage, neurotoxicity, apoptosis, and immunotoxicity. This finding suggests that short-chain PFAS may cause substantial physiological disturbance under certain exposure conditions [76]. These exceptions indicate that PFAS toxicity is not determined by the chain length alone. Food-web transfer and exposure conditions affect the dose received; the uptake and tissue distribution determine the internal exposure; and receptor activation and target-organ sensitivity govern the resulting biological effects. Therefore, these factors should be considered together in ecological risk assessment and environmental management to avoid misleading conclusions based on a single endpoint.

## 5. PFAS with Different Functional Groups

### 5.1. Physicochemical Properties with Different Functional Groups

PFAS mainly include PFCAs, PFSAs, perfluoroalkane sulfonamides (FASAs), fluorotelomer-based compounds (FTCs), and emerging ether-based PFAS, including per- and polyfluoroether carboxylic acids (PFECAs) and per- and polyfluoroether sulfonic acids (PFESAs) [77]. Among these compounds, PFCAs and PFSAs are the terminal degradation products most frequently detected in the environment, whereas fluorotelomer alcohols (FTOHs) and FASAs can serve as precursors that are further transformed into PFCAs or PFSAs [1]. Due to differences in the functional groups, distinct physicochemical properties are exhibited by different PFAS. In conventional PFAS, PFCAs contain a carboxylate group (–COO^−^) as the polar head group, whereas PFSAs contain a sulfonate group (–SO_3_^−^). Because the sulfonate group has stronger acidity and polarization capacity, PFSAs generally exhibit higher surface activity and stronger protein-binding affinity than PFCAs with the same carbon-chain length [78,79]. Compared with PFCAs and PFSAs, fluorotelomer sulfonates (FTSs) and sulfonamide-based PFAS, such as FASAs, show more complex environmental behaviors. FTSs usually have relatively high volatility and can be transported over long distances through the atmosphere to remote regions, where they may be gradually oxidized and transformed into stable PFCAs [80]. In contrast, the mobility of FASAs is generally lower because of their partially neutral molecular characteristics. Emerging ether-based PFAS, such as GenX, ADONA, and 6:2 Cl-PFESA, have been developed by introducing ether bonds into the perfluorocarbon chain, thereby altering the molecular structures of conventional PFAS. The introduction of ether bonds increases the molecular flexibility and water solubility and may reduce the bioaccumulation potential of traditional long-chain PFAS to some extent. However, this structural modification also changes their environmental transport behavior and toxicokinetic properties. Although some ether-based PFAS exhibit lower bioaccumulation potential, high environmental persistence and potential ecological risks have still been observed [81]. Overall, the type of functional group not only determines the physicochemical properties of PFAS but also may further influence their bioaccumulation potential and ecotoxicity.

### 5.2. Bioaccumulation and Distribution with Different Functional Groups

Functional-group identity is an important factor controlling PFAS bioaccumulation. Under comparable structural backbones, identical species, and similar aqueous exposure conditions, the bioaccumulation potential of PFAS with different functional groups can generally be ranked as follows: FASAs >PFSAs > PFCAs, whereas the FTSs and perfluoroether substances vary considerably [82]. The bioconcentration factor (BCF) is defined as the ratio of the PFAS concentration in an organism or a specific tissue to that in the water and reflects the chemical uptake from the aqueous phase in the absence of dietary exposure. In fathead minnows (*Pimephales promelas*) continuously exposed to contaminated groundwater, the mean BCF of perfluorooctanesulfonamide (FOSA) in the kidney reached 8700 L/kg, which was the highest value among all target PFAS in that study. By contrast, the BCFs of PFOS in the liver, kidney, and gonads were approximately 730, 490, and 180 L/kg, respectively, indicating that FASAs represented by FOSA have a substantially stronger tissue-concentration capacity than PFSAs [83]. Furthermore, the BAF of PFOS was approximately 2–3 times higher than that of PFOA, suggesting that PFSAs generally have a higher bioaccumulation potential than PFCAs with similar perfluorocarbon backbones [84,85]. This difference has also been observed in amphibians [20]. In a 40 d aqueous exposure study of northern leopard frog tadpoles (*Lithobates pipiens*), Hoover et al. compared the accumulation of PFOS, PFOA, and 6:2 FTS. The whole-body BCF of PFOS ranged from 19.6 to 119.3, whereas the BCFs of 6:2 FTS and PFOA remained below 1. These results showed that, in the same species, exposure duration, and nominal exposure level, the internal concentration of 6:2 FTS was much lower than that of PFOS [86]. Ether-based PFAS should not be regarded simply as low-bioaccumulation compounds because of the presence of ether bonds. For example, the whole-body BAF of the ether sulfonate substitute F-53B in *crucian carp* was significantly higher than that of PFOS in the same dataset, indicating that F-53B still has strong bioaccumulation potential [87].

PFAS with different functional groups also exhibit organ-specific accumulation patterns. In the same fathead minnow study, the highest BCF of FOSA was detected in the kidney, whereas the highest BCF of PFOS was observed in the liver. This suggests that sulfonamide-based PFAS may be more prone to renal accumulation, whereas sulfonate-based PFAS tended to maintain higher concentrations in the liver [83]. A rainbow trout (*Oncorhynchus mykiss*) study also showed that PFAA distribution was mainly concentrated in the blood, liver, and kidney, although the relative proportions of compounds with different functional groups varied among these tissues. Therefore, when the effects of functional groups on PFAS bioaccumulation are evaluated, whole-body BCFs or BAFs should be assessed together with concentrations in key tissues, such as the liver, kidney, and blood, which can help avoid inaccurate conclusions based only on the concentrations in a single tissue [85].

The bioaccumulation potential of PFAS with different functional groups is jointly regulated by their binding affinities for plasma proteins and phospholipids, as well as by renal transport and clearance processes. Functional-group identity determines the binding capacity of PFAS with biological macromolecules. Because of its stronger polarity and charge distribution, the sulfonate group can form more stable complexes with proteins, thereby enhancing the retention of PFAS in blood and liver. In human blood samples donated by more than 600 adults, PFHxS, PFOS, PFOA, and PFNA were detected at 2.3, 35.1, 4.7, and 0.6 ng/mL, respectively [88]. Differential scanning fluorimetry has been used to characterize the dissociation constant (Kd) values of different PFAS with human serum albumin (HSA), and the binding affinity was ranked as follows: sulfonates > carboxylates > ether acids [89]. Differences in the binding affinity may also affect PFAS transport and excretion, thereby influencing bioaccumulation. Little metabolism of PFAS occurs in organisms, and their elimination is mainly dependent on renal transporter-mediated urinary excretion. PFCAs generally have a relatively high renal clearance efficiency, whereas PFSAs bind more strongly to renal tubular transporters and tissue proteins. As a result, longer biological half-lives and higher tissue retention are observed for PFSAs [56]. In addition, these binding differences are closely related to organ-specific characteristics. Higher accumulation in the liver has been associated with the strong affinity of liver fatty acid-binding proteins for PFAS, whereas gonads contain abundant phospholipids that can promote PFAS binding [90]. These toxicokinetic processes are key determinants of the tissue-specific distribution of PFAS with different functional groups. However, caution is warranted when extrapolating these findings to aquatic organisms. Interspecies differences in plasma protein composition, renal physiology, transporter expression, and gill-mediated elimination may substantially influence the distribution, clearance, and bioaccumulation of PFAS.

### 5.3. Trophic Magnification Associated with Different Functional Groups

**Figure 5:**
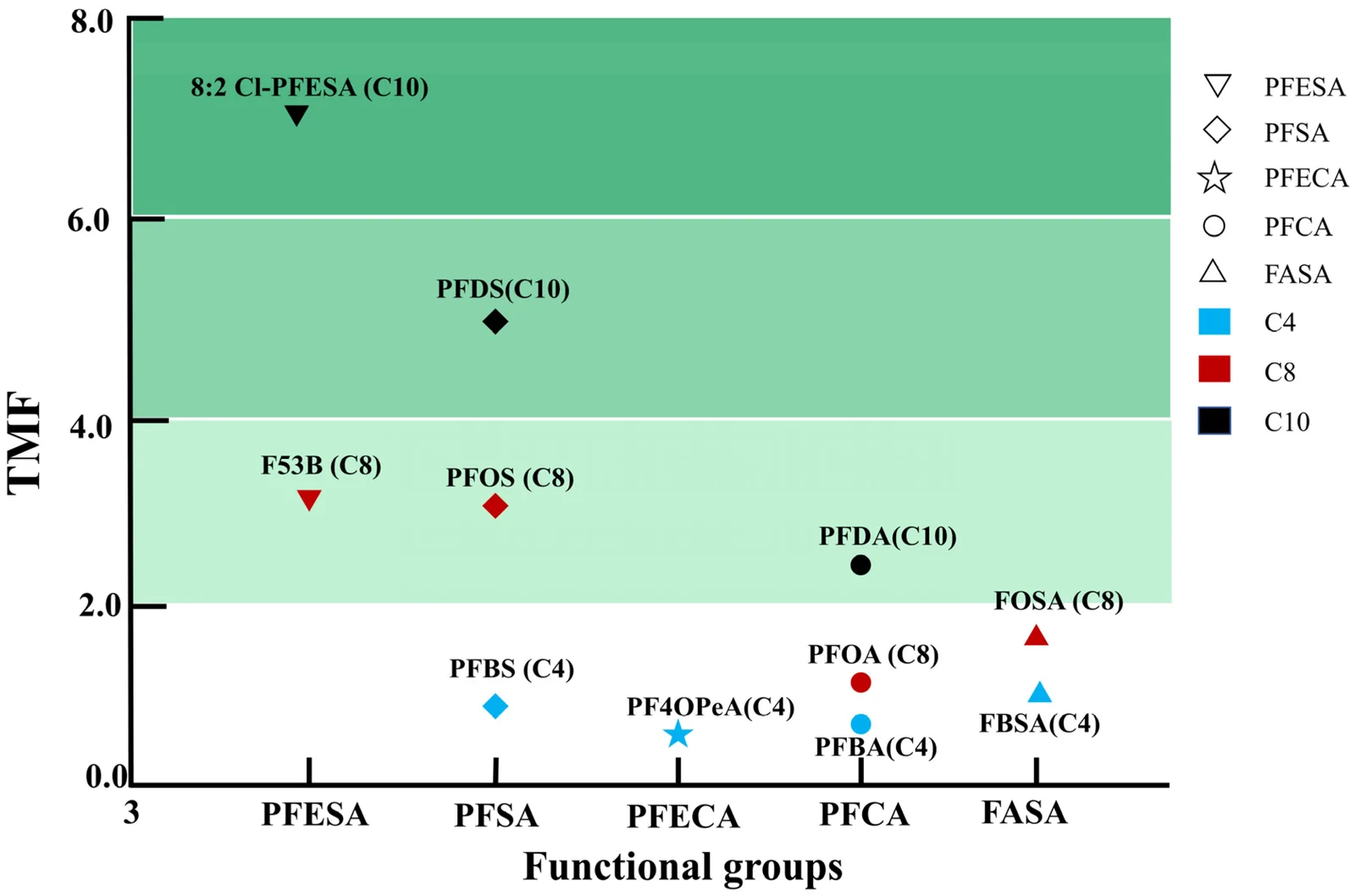
Trophic magnification factors (TMFs) of per- and polyfluoroalkyl substances (PFAS) with different functional groups in aquatic food webs.

Differences in bioaccumulation caused by functional groups can also affect the trophic magnification behavior of PFAS (Figure 5). At the same or similar chain lengths, PFSAs generally exhibit stronger trophic magnification potential than PFCAs. For example, stable biomagnification of PFOS has often been reported in aquatic food webs, with a TMF of 4.5, whereas PFOA shows much lower trophic magnification potential, with a TMF of 1.14, or even trophic dilution, with a TMF of 0.34 [63,91,92]. Thus, clear biomagnification of PFCAs is usually observed only when the carbon chain is longer. More complex TMF patterns have been observed for FASAs. As typical precursor PFAS, their TMFs may reflect not only their own accumulation potential but also precursor transformation processes. For example, FOSA can be biomagnified in food webs and may serve as a precursor source for PFOS formation in organisms at higher trophic levels [92,93]. In contrast, some FASA or FTS precursors, such as N-EtFOSAA and 6:2 FTSA, have been detected at low frequencies or have not shown significant trophic magnification in riverine food webs. This indicates that metabolizable precursor PFAS do not necessarily share the same TMF characteristics as stable terminal products [91,94]. Ether-containing alternative PFAS also show notable differences in TMFs. Some chlorinated PFESAs have been reported to exhibit high trophic magnification potential in multiple aquatic food-web studies and have been identified as PFAS with relatively high TMFs, which suggests that the presence of ether structures does not necessarily reduce the capacity of PFAS to be magnified along food webs [95,96]. For example, a meta-analysis using a unified analytical framework reported a slightly higher pooled TMF for F-53B (TMF = 3.07) than for PFOS (TMF = 3.02) [93]. Similar patterns have also been observed for PFECAs. In a source-impacted estuarine food web, perfluoro-3,5,7,9,11-pentaoxadodecanoic acid (PFO5DoA, C7) and hexafluoropropylene oxide trimer acid (HFPO-TA, C9) showed TMFs of 5.62 and 5.52, respectively, indicating substantial trophic magnification despite their relatively short perfluorinated carbon chains [66]. These findings suggest that the incorporation of ether linkages does not necessarily reduce the trophic magnification potential of PFAS. Note that between-study differences in reported TMFs may reflect not only compound-specific properties but also methodological variability, which may be a major source of TMF variation [93]. Future studies should clearly define food-web boundaries and contamination sources, apply consistent stable-isotope approaches and appropriate baseline organisms for trophic-level assignment, and standardize the types of biological samples collected across trophic levels. Concentrations should be reported on a clearly specified wet- or dry-weight basis, together with the relevant conversion factors, and both protein-normalized and non-normalized concentrations should be provided. In addition, the treatment of nondetects, precursor transformation, sample size, regression models, and confidence intervals for TMF estimates should be fully documented and incorporated into standardized methodological frameworks.

### 5.4. Toxicity Differences with Different Functional Groups

Under the same or similar carbon-chain lengths, significant differences in toxicity have been observed among PFAS with different functional groups. Overall, the toxicity is generally ranked as PFSAs > PFCAs. The introduction of ether bonds is commonly considered to reduce the bioaccumulation potential and thereby weaken the toxicity. Such differences have been reflected across multiple toxicological endpoints, including hepatotoxicity [20,97], neurotoxicity [98,99], and immunotoxicity [29].

Interestingly, opposite patterns have been observed in some invertebrates. Under exposure to four common PFAS, namely PFOS, PFOA, PFNA, and PFDA, higher disruption of lysosomal stability was induced by PFCAs in the green mussel (*Perna viridis*), whereas only slight adverse effects were caused by PFOS [100]. In addition, the PFOS substitute F-53B generally shows stronger hepatotoxicity and immunotoxicity. By contrast, PFOA and its emerging alternatives, HFPO-DA and HFPO-TA, were all shown to induce intestinal inflammation and apoptosis in zebrafish, with comparable toxic effects among the three compounds [101]. Overall, PFSAs usually exhibit the highest toxicity, whereas carboxylates, ether acids, and some alternative PFAS show relatively lower toxicity. The stronger binding of PFSAs to plasma proteins and cell membranes may increase accumulation and target-organ exposure. Separately, functional-group-dependent interactions with nuclear receptors, membrane proteins, and signaling pathways may alter lipid metabolism, neurotransmission, and immune-inflammatory responses [102]. Although structural modifications such as ether-bond introduction may reduce the bioaccumulation potential of some PFAS, molecular conformation and receptor-binding patterns can also be changed, leading to altered toxicity profiles [103]. Moreover, the toxicity may also be closely associated with species-specific bioaccumulation capacity and exposure routes.

Overall, the functional-group identity plays an important role in determining the bioaccumulation, tissue distribution, trophic magnification, and toxicity of PFAS in aquatic organisms. PFSAs generally exhibit stronger protein binding, bioaccumulation, and toxic effects than PFCAs, and some sulfonamide- and ether-containing PFAS may show distinct accumulation or toxicity profiles. Therefore, PFAS alternatives should not be assumed to be environmentally safer solely on the basis of functional-group modification.

## 6. PFAS in Acid and Salt Forms

### 6.1. Physicochemical Properties of PFAS in Acid and Salt Forms

Aside from differences in their chain length and functional groups, PFAS can occur in diverse chemical forms in environmental and biological systems [104]. Specifically, the acid form generally refers to the protonated electrically neutral molecule, whereas the salt form consists of a deprotonated PFAS anion paired with counterions such as Na^+^, K^+^, or NH_4_^+^. The relative proportions of these two forms in the aqueous phase are mainly determined by the environmental pH and the pKa of the compound [105]. Because PFCAs and PFSAs are generally strong acids, they are predominantly present as deprotonated anions under most natural water and physiological conditions [106,107]. Neutral acid-form PFAS are less hydrated and usually exhibit higher vapor pressures and stronger air–water partitioning tendencies. Therefore, they are more likely to migrate across the water–air interface or enter the gas phase [108]. In contrast, salt-form PFAS are charged and can form ionic lattices, resulting in higher apparent water solubility and markedly lower volatility. As a result, they are more readily retained in the aqueous phase and transported with water. After dissociation in water, salt-form PFAS still retain the hydrophobic and oleophobic properties derived from the perfluorocarbon chain. These physicochemical differences may alter their environmental availability and uptake, thereby affecting internal exposure and observed toxicity.

### 6.2. Bioaccumulation and Toxicity of PFAS in Acid and Salt Forms

Multiple studies have consistently shown that acid-form PFAS exhibit stronger bioaccumulation potential than their salt-form counterparts. In an acute zebrafish embryo exposure study, acid forms of PFOA, PFBA, and PFBS were accumulated at significantly higher levels than their corresponding salt forms [109]. These differences in accumulation may be an important driver of toxicity variation. Acid-form PFAS significantly increased the mortality and malformation rates in zebrafish embryos and inhibited their heart rate, growth, and development, with markedly higher overall toxicity than that of salt-form PFAS. For example, exposure to 467.2 μM PFOA acid caused complete mortality in zebrafish larvae, whereas only 17.3% mortality was induced by PFOA salt at the same concentration [109]. At the mechanistic level, stronger disruption of the thyroid axis was also induced by acid-form PFAS, as reflected by altered T3/T4 levels and the abnormal expression of downstream genes. Proteomic analysis further showed that a much larger number of differentially expressed proteins was induced by acid-form PFOA than by its salt form. Pathways related to myocardial contraction, vascular regulation, and adrenergic signaling were significantly affected, leading to more severe developmental cardiotoxicity [110]. Differences between PFAS acid and salt forms in toxicity and bioaccumulation can also be transmitted across generations. Long-term exposure studies in adult zebrafish showed that acid-form PFAS, including PFOA, PFBA, and PFBS, were accumulated at significantly higher levels in both parental and offspring generations than their salt-form counterparts; hence, stronger intergenerational transfer was observed. In addition, more pronounced hypothyroidism was induced by acid-form PFAS, and stronger endocrine-disrupting effects and intergenerational toxicity were observed in both parental and offspring generations. These findings suggest that the long-term ecological risks of acid-form PFAS may be substantially higher than those of their salt forms [111].

The toxicity differences between PFAS acid and salt forms may arise from toxicokinetic processes. First, stronger binding to transport proteins, such as serum albumin, has been observed for acid-form PFAS, which may prolong biological half-lives and increase target-organ exposure. For example, biolayer interferometry (BLI) analysis showed that the dissociation constant (KD) of acid-form PFOA for serum albumin was 29.9 μM, whereas that of salt-form PFOA was 45.4 μM. Similarly, among sulfonates, the KD of PFBS acid was 96 μM, whereas no binding was detected for PFBS salt [109]. This difference may be mainly attributed to the stabilization of noncovalent interactions between long-chain fluorinated compounds and albumin under acidic conditions [54,79,112,113]. Similarly, differences in binding affinity for nuclear receptors may contribute to the toxicity variation. For example, a higher binding affinity for thyroid hormone receptor β (TRβ) has been reported for acid-form PFAS, allowing transcriptional activity to be inhibited more effectively and downstream toxic effects to be triggered [111]. Higher bioaccumulation may increase internal exposure, whereas broader pathway perturbations may increase the severity of systemic effects. Overall, nominal acid-form PFAS may produce higher internal exposure because of stronger protein binding and may exert stronger biological effects through altered receptor interactions. However, the nominal acid and salt forms used in exposure experiments may not correspond to the stable species present under environmentally relevant conditions; this limitation should be carefully considered when comparing their environmental risks.

## 7. Conclusions and Perspectives

This review focused on the toxic effects of PFAS on aquatic animals and their structure-dependent differences. The bioaccumulation, distribution, biomagnification, and toxicity of PFAS in aquatic organisms were critically synthesized and compared. The current evidence indicates that the aquatic ecological risks of PFAS are characterized by multidimensional structure-dependent differences. PFAS with long carbon-chain backbones, sulfonate groups, or acid forms should therefore be prioritized in ecological risk assessment.

To improve the understanding of the environmental risks posed by PFECAs and to support the development of sustainable alternatives, future studies should focus on the following areas.

(1) A structure-dependent framework for aquatic ecological risk assessment of PFAS should be established. PFAS bioaccumulation and toxicity are strongly affected by the chain length, functional group, and chemical form. However, current risk assessments are often based on single representative compounds or total PFAS concentrations that do not accurately reflect the risk differences among structural classes. A more refined structure-dependent assessment system should therefore be developed. Key parameters, including carbon-chain length, functional-group type, acid–salt form, protein-binding affinity, phospholipid partitioning, renal clearance rate, and biological half-life, should be incorporated into integrated analyses. In addition, bioaccumulation patterns and toxicity modes should be differentiated between functional groups. Such a framework would improve the scientific basis for PFAS alternative screening and aquatic ecological risk management.
(2) Second, studies on environmentally realistic mixture exposure and food-web transfer should be strengthened. In natural waters, aquatic organisms are exposed to complex mixtures of legacy PFAS, emerging PFAS, precursors, and transformation products. Interactions among different PFAS may occur during uptake, protein binding, metabolic transformation, excretion, and toxic pathway activation, resulting in additive, synergistic, or antagonistic effects. Future studies should therefore be conducted more frequently under environmentally relevant concentrations and realistic contamination scenarios. Stable isotope-based trophic level analysis, TMF/BAF/BCF calculations, and multi-tissue distribution data should also be integrated to clarify the PFAS transfer among plankton, benthic invertebrates, fish, amphibians, and higher-trophic-level organisms. This information is critical for identifying high-risk compounds and key exposure pathways in aquatic food webs.
(3) Third, more comparable data across species, tissues, and chemical forms should be generated. Current studies on PFAS bioaccumulation and toxicity differ substantially in their experimental species, exposure duration, concentration settings, target tissues, and toxicological endpoints, which limits a direct comparison across studies. Future assessment frameworks should therefore be further standardized by clearly reporting chemical forms, exposure-medium concentrations, wet-weight/dry-weight conversions, whole-body and tissue-specific BCFs/BAFs, elimination half-lives, and key toxicological endpoints. By improving data comparability, the relationships between PFAS structural characteristics and aquatic ecological risks can be identified more accurately, thereby providing a more reliable basis for environmental standard development and alternative safety assessment.

## Figures and Tables

**Table 1:** Relative responses of human peroxisome proliferator-activated receptor alpha (PPARα) to perfluoroalkyl acids (PFAAs) in transiently transfected COS-1 cells, measured by C_20max_, from most active to least active [71].

Compound	Carbon Number	C_20max_ (μM)
PFBA	4	75
PFPeA	5	52
PFHxA	6	47
PFHpA	7	15
PFOA	8	16
PFNA	9	11
PFDA	10	NA
PFDoA	12	NC

C_20max_: the concentration required for a perfluoroalkyl acid (PFAA) to induce 20% of the maximal response; a lower value generally indicates a stronger activating effect; NA, not active; NC, could not calculate a value.

## Data Availability

The raw data supporting the conclusions of this article will be made available by the authors on request.
